# Biomechanical effects of foot orthoses with and without a lateral bar in individuals with cavus feet during comfortable and fast walking

**DOI:** 10.1371/journal.pone.0248658

**Published:** 2021-03-17

**Authors:** Gabriel Moisan, Martin Descarreaux, Vincent Cantin

**Affiliations:** 1 Department of Human Kinetics, Université du Québec à Trois-Rivières, Trois-Rivières, PQ, Canada; 2 Groupe de Recherche sur les Affections Neuro-musculo-squelettiques (GRAN), Université du Québec à Trois-Rivières, Trois-Rivières, PQ, Canada; West Park Healthcare Centre, CANADA

## Abstract

**Background/purpose:**

The biomechanical effects of foot orthoses (FOs) with and without a lateral bar compared to a control condition during walking at different speeds are still unknown. The objective of this study was to compare the biomechanical effects of functional FOs with and without a lateral bar to a control condition during comfortable walking in individuals with cavus feet and determine if their effects change at a fast speed.

**Methods:**

Fifteen individuals with cavus feet (age: 25.3 ± 5.8 yrs) walked under two experimental conditions (FOs with and without a lateral bar) and a control condition (shoes only) at comfortable (CW) and fast (FW) speeds. The outcome measures were ankle and knee angles and gluteus medius, vastus lateralis, gastrocnemius lateralis, gastrocnemius medialis, peroneus longus and tibialis anterior electromyography (EMG) amplitudes during the stance phase of walking and were compared between the FOs and a control condition using one-dimensional statistical parametric mapping.

**Results:**

During CW, both FOs decreased ankle dorsiflexion and increased knee extension angles compared to no FOs. FOs with a lateral bar also decreased peroneus longus EMG amplitudes. During FW, FOs with and without a lateral bar decreased ankle dorsiflexion angles compared to no FOs.

**Conclusion:**

Both types of FOs had different effects on the biomechanics of the lower limb compared to a control condition. The decreased peroneus longus EMG amplitudes during CW in individuals with cavus feet could have important clinical implications in other populations, such as individuals with painful cavus feet. The orthoses only affected the ankle dorsiflexion angles at a fast speed and no EMG amplitude or knee kinematics effects were observed. Further studies assessing the ankle kinematics and kinetics effects of these orthoses are needed to improve our understanding of their mechanism of action and inform future efficacy trials.

## Introduction

Foot orthoses (FOs) are prescribed to treat and prevent many lower limb musculoskeletal conditions [1, 2]. FOs with different geometries (e.g. arch supports) or extrinsic modifications (e.g. rearfoot posts) can change rearfoot [3, 4], tibia [4] and knee [5] kinematics during walking, but these differences are generally small (i.e. few degrees of changes) and may only partially explain the FOs’ beneficial effects (e.g. pain reduction) in the treatment of musculoskeletal pathologies [6]. FOs can also increase knee external rotation [4, 7] and adduction [7] external moments, increase knee abduction internal moments [8], and decrease ankle eversion external moments [4]. FOs affect the lower-limbs’ distal joints in individuals with flat feet [9] but it is unknown if these results are generalizable to individuals with different foot types. Previous studies also showed decreased tibialis posterior [10] and tibialis anterior [11] and increased peroneus longus and gastrocnemius lateralis [12] electromyography (EMG) amplitudes when wearing FOs during walking. Previous studies investigating the biomechanical effects of FOs mostly involved participants with flat or rectus feet [6, 9] and it is unclear whether the observed changes in muscle function using FOs are consistent and predictable across foot morphologies [13].

The prevalence of cavus feet in the population can be as high as 15% [14] and individuals with this foot type present biomechanical differences compared to counterparts with flatter feet during walking, such as decreased peak rearfoot eversion [15], lateralized ground reaction forces [16] and increased pressure under the rearfoot [17]. Little is known about the effects of FOs in individuals with cavus feet. Better understanding the effects of FOs for these individuals is essential to inform treatment targeted to their particular biomechanics. In clinical contexts, these biomechanical differences are considered during the fabrication of custom FOs or the choice of prefabricated FOs in order for them to be as patient specific as possible. However, most FOs used in previous studies are generic and have little customization to the participants’ particular biomechanics, limiting application of the published research in clinical practice. One way of making FOs more patient specific is to add extrinsic modifications, such as a lateral bar, to the FOs’ shell. The aim of adding lateral bars to FOs is to limit ankle inversion motion and external inversion moments during locomotion [11, 12], which are known to be increased in individuals with cavus feet [15–17]. As a result, adding a lateral bar to FOs could consequently decrease the pronator muscles activity. In fact, FOs with a lateral bar decreased the EMG amplitudes of the peroneus longus and gastrocnemius lateralis compared to a control condition [11], while the same FOs without the lateral bar decreased the tibialis anterior [11] and increased the gastrocnemius lateralis [12] EMG amplitudes during the stance phase of walking in individuals with rectus and cavus feet. However, the effect of adding this bar on the kinematics of the lower limb is still unknown. In fact, one of the main limitation of previous studies assessing the effects of FOs on the biomechanics of the lower limbs is the lack of concurrent EMG, kinematic and/or kinetic investigation [18]. FOs with a lateral bar can be used in clinical contexts to modify the biomechanics of the lower limb of individuals with musculoskeletal pathologies, such as chronic ankle instability [19]. However, clinical studies are needed to determine if the biomechanical effects of FOs with a lateral bar will translate into clinical improvements for this population.

Most previous studies that quantified the effects of FOs on lower-limb biomechanics had the participants walk at a comfortable self-selected speed [3, 4, 7, 8, 10, 11, 19, 20], whereas one study had them walk at a very fast speed [12]. When walking speed increases, lower-limb biomechanics change [21–23], but no study has yet investigated if the biomechanical effects of FOs change accordingly. As the biomechanical demand to the musculoskeletal system is increased at a faster walking speed, one may rely more on the FOs to assist locomotion and thus, greater effects could perhaps be observed.

Therefore, the objectives of this study were to compare the kinematic and EMG effects of FOs with and without a lateral bar with a control condition (FOs with a lateral bar vs shoes only and FOs without a lateral bar vs shoes only) during walking in individuals with cavus feet and determine if their effects change at a fast speed. The main hypotheses were that the EMG amplitudes of the pronator muscles and ankle dorsiflexion angle will be decreased with FOs with a lateral bar and increased with FOs with no bar compared to a control condition. Also, these changes will be more pronounced at a fast speed.

## Materials and methods

### Participants

Fifteen healthy participants were recruited among the Université du Québec à Trois-Rivières (UQTR) students and from the UQTR outpatient podiatry clinic. To be characterized as having cavus feet, the participants’ FPI [24] score, calculated by the same researcher, had to be -2 or less for at least one foot. This threshold was used in previous studies [12, 25] and was chosen to increase the external validity of our results. The exclusion criteria were having painful feet, congenital cavus feet or any condition known to adversely affect gait and having worn FOs within the last three months. The UQTR Ethics Committee granted ethical approval for this study, and participants gave written informed consent before their involvement (CER-14-199-07-17).

### Foot orthoses

**Fig 1:**
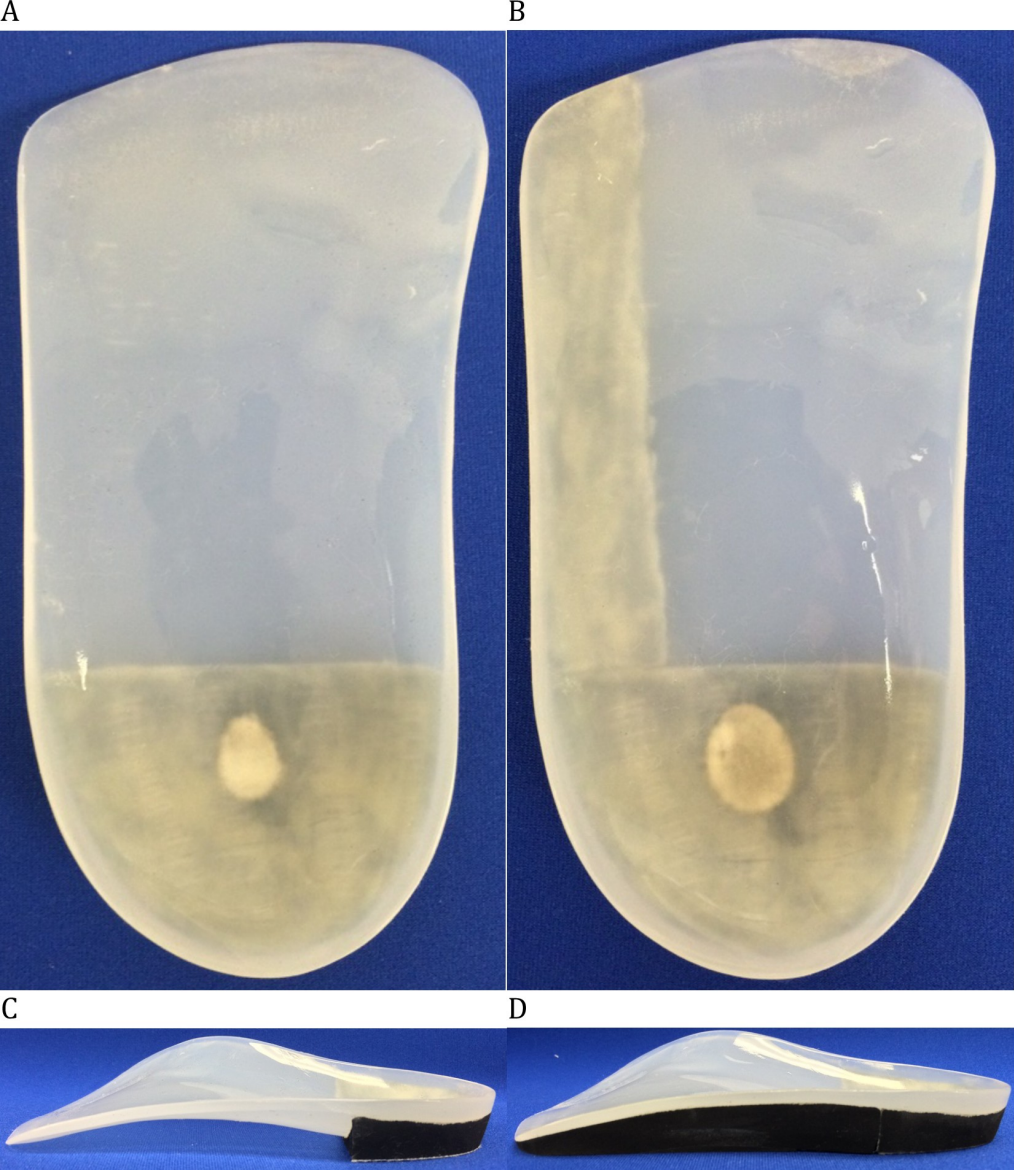
(A) Foot orthoses (top) (B) Foot orthoses with a lateral bar (top) (C) Foot orthoses (side) (D) Foot orthoses with a lateral bar (side).

All participants had negative plaster casts taken by the same licensed podiatrist (GM), with the subtalar joint held in neutral position with the participant in supine position [26]. The same certified orthotic technician produced the positive casts and molded the FOs on them. Minimal arch fills were used. FOs were made of a 3.2 mm thick polypropylene shell, cut proximal to the metatarsal heads. A straight extrinsic ethylene-vinyl-acetate (EVA, Durometer: 55) rearfoot post, commonly used in clinical practice, was glued under the 14 mm heel cup and a lateral bar was glued under the lateral part of the FOs in the gap between the rearfoot post and the anterior edge (See Fig 1). Then, the lateral bar was ground in order for it to be leveled with the rearfoot post. The bar could be removed, when needed, by heating it with a heat gun and added to the FOs with contact glue. During the experimental sessions, all participants wore the FOs in the same shoe model (Athletic Works, Model: Rupert, Bentonville, AR, USA). Between the sessions, the participants were asked to wear the orthoses in their own shoes.

### Instrumentation

All participants underwent two clinical gait analyses (with and without orthoses) during which kinematic and EMG data of the lower limb with the lowest FPI score (or the dominant leg when equal) were collected. Kinematic data were recorded at a sampling rate of 100 Hz with a three-dimensional motion analysis system including nine cameras (Optotrak Certus, Northern Digital, Waterloo, Ontario, Canada). A modified version of the validated cluster-based conventional gait model [27] was used. Kinematic markers were positioned on the tested limb on the greater trochanter, lateral femoral epicondyle, lateral malleolus and fifth metatarsal head (on the shoe). To create virtual markers on the medial femoral epicondyle and medial malleolus, a digitizing probe was used. Clusters of three non-colinear markers were positioned on the distal 1/3 of the lateral part of the thigh and the lower leg. Ground reaction forces were collected at a sampling rate of 1000 Hz with a force platform (Bertec Corp, Columbus, OH, USA) embedded in the floor. The vertical ground reaction forces were used to detect the initial contact and the toe off events using a 10 N threshold. Walking speed was monitored with electronic photocells timing gates (Brower Timing System, Draper, UT, USA) positioned 1.35 m before and after the force platform.

Surface EMG data were collected using single differential Ag electrodes (Model DE2.1, Delsys Inc., Boston, MA, USA) applied over the gluteus medius, vastus lateralis, gastrocnemius lateralis and medialis, peroneus longus, and tibialis anterior muscles according to the recommendations of SENIAM [28]. These muscles are commonly assessed in gait analysis with and without FOs and represent muscle groups highly solicited during walking [13, 18]. To reduce impedance, the skin was shaved, abraded with fine-grade sandpaper and then wiped with alcohol swabs. A reference electrode was placed over the ipsilateral anterior superior iliac spine. EMG signals were differentially amplified (AMT-8, common mode rejection ratio of 92 dB at 60 Hz, input impedance of 10 GW; 12-bit A/D converter) and sampled at 1000 Hz.

### Protocol

Prior to the first experimental session, participants had to wear the orthoses during their daily activity for one month. Eight participants were randomly given FOs with a lateral bar and seven were given FOs with no bar using a random number table. All participants had to complete an adaptation protocol consisting of adding one hour of wear per day until they could wear them comfortably all day. All participants were asked to complete a daily logbook in which they had to record the number of hours they wore their orthoses. They were asked to wear the orthoses for at least five hours per day on average.

Two identical experimental sessions (except for the worn orthoses) were undertaken one month apart. The experimental protocol consisted of walking on a 5-meter walkway under two experimental conditions (with and without the orthoses) and two walking speeds (comfortable (CW) and fast (FW)). The FW speed was described to the participants as the fastest they could walk without running. The order of all test conditions and speeds were randomized across participants using a random number table and the same order was used for the second testing session. Prior to the walking trials, a calibration trial was recorded in order to create the thigh/leg/foot segments and calculate hip/knee/ankle joint centers. To familiarize themselves with the experimental protocol, all participants were instructed to walk six times on the walkway, using a midgait protocol (the force platform was located halfway on the participants’ path), prior to each test condition and speed. During these trials, mean walking speed was recorded, averaged and used as a reference for the recorded trials. Six recorded trials were performed for both experimental conditions and speeds. A trial was rejected and immediately retaken if speed varied ±5% of the predetermined mean speed.

After the first testing session, the experimental conditions were interchanged for all participants: a lateral bar was added to the FOs that did not have one and removed from those that previously had a lateral bar. All participants wore the new orthoses in their everyday activities for the next month after undergoing the same adaptation protocol and subsequently completed the second testing session during which the same protocol was performed.

#### Data processing

Kinematic data were exported into Visual3D software (C-motion, Germantown, MD, USA) and were low-pass filtered by a dual-pass, fourth-order Butterworth filter with a cut-off frequency of 6 Hz and 50 Hz, respectively. Three-dimensional joint angles were calculated with a Cardan sequence of X (extension/flexion), Y (adduction/abduction), and Z (internal/external rotation). Technical limitation of the volume of capture restrained the possibility to place three non-collinear markers on the shoe. Thus, only the sagittal plane ankle angle (X) was calculated during the walking trials and was normalized to the static trial ankle angle. EMG data were digitally filtered with a zero-phase lag, bidirectional, 10 Hz to 450 Hz bandpass fourth-order Butterworth filter using a custom MATLAB file (Mathworks, Inc., Natick, MA, USA). Root Mean Square (RMS) of these data, calculated with a 25-ms moving window, was used for the analyses. RMS data of each muscle were normalized with the mean peak RMS amplitude of all trials of the control condition (shod without FOs) at fast walking speed.

### Analysis

The normality of the walking speed and biomechanical data was evaluated with Shapiro-Wilk and D’Agostino-Pearson tests, respectively. Dependent t-tests were used to compare walking speed of each experimental condition with their respective control condition as the data were normally distributed. The biomechanical data of each individual stance phase was normalized to 100%. As the kinematic data were normally distributed, dependent t-tests (SPM(t)) [29] were used to compare each normalized point of the curves in Python software (Version 2.7). As EMG data were not normally distributed, non-parametric permutation tests (SnPM) [30] were used. The SPM(t) and SnPM thresholds above which only α = 5% of the data would be expected, had the test statistic trajectory resulted from an equivalently smooth random process, were calculated. The individual probability that each supra-threshold cluster could have resulted from an equivalently smooth random process was determined. Due to poor between-session absolute reliability of the biomechanical outcomes during walking, especially EMG [11], only within-session comparisons (orthoses with their respective control condition) were performed and no comparisons were made between both types of FOs.

## Results

Six men and nine women (age: 25.3 ± 5.8 yrs., height: 170.9 ± 10.6 cm, weight: 68.6 ± 15.2 kg, Foot Posture Index (FPI) score: -4.9 ± 2.4) were recruited in this study. The number of hours the experimental conditions were worn during the adaptation periods were 5.6 ± 1.1h/day for FOs and 5.4 ± 1.7h/day for FOs with a lateral bar.

### Walking speed

Walking speed was decreased during walking with FOs with a lateral bar compared to the respective control condition at CW (1.45 ± 0.21 vs. 1.49 ± 0.23 m/s, *P* = 0.05). No significant difference was found for FOs with a lateral bar with the respective control condition at FW (2.18 ± 0.23 vs. 2.22 ± 0.26 m/s, *P* = 0.12) and FOs compared with the respective control condition (CW = 1.39 ± 0.15 vs. 1.40 ± 0.17 m/s, *P* = 0.48, FW = 2.20 ± 0.25 vs. 2.21 ± 0.17, *P* = 0.51).

### Biomechanical data

#### FOs (without a lateral bar)

**Fig 2:**
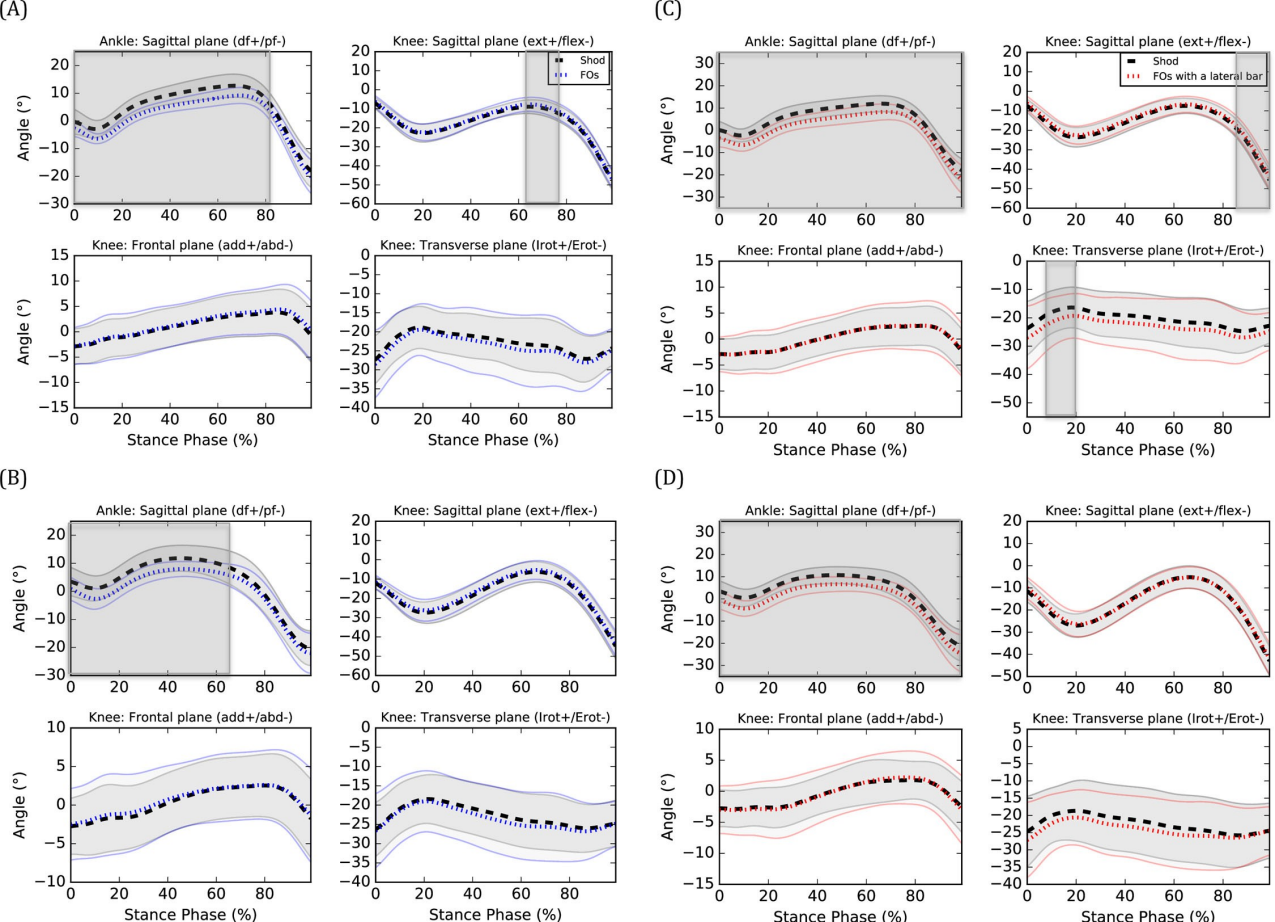
Kinematic effects of (A) FOs at CW, (B) FOs at FW, (C) FOs with a lateral bar at CW and (D) FOs with a lateral bar at FW. ext: extension, flex: flexion, add: adduction, abd: abduction, Irot: internal rotation, Erot: external rotation. Means of the shod (black), FOs (blue) and FOs with a lateral bar (red) are represented by dotted lines and standard deviations are observed between the full lines. Significant between-group differences are observed in the shaded region.

**Fig 3:**
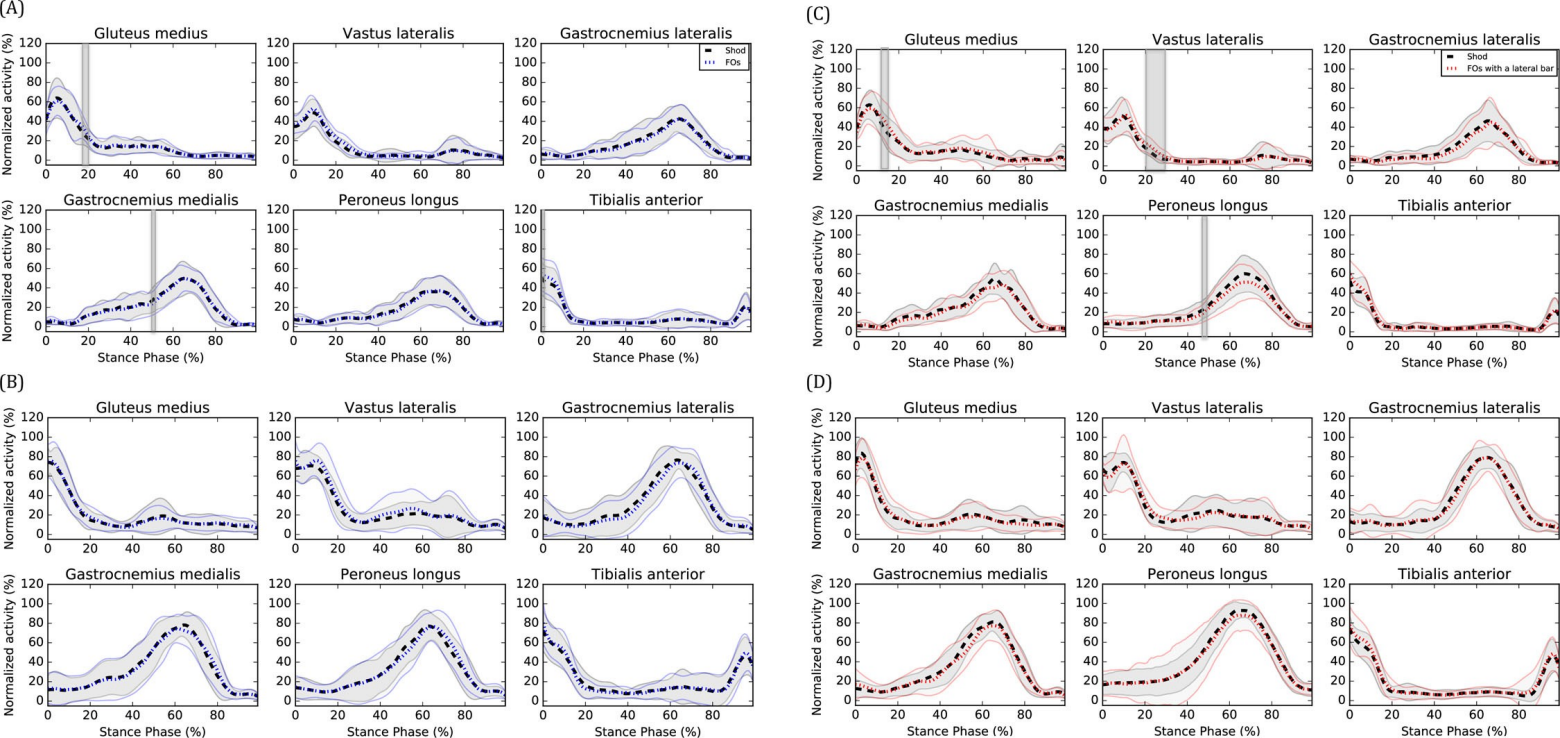
EMG effects of (A) FOs at CW, (B) FOs at FW, (C) FOs with a lateral bar at CW and (D) FOs with a lateral bar at FW. Means of the shod (black), FOs (blue) and FOs with a lateral bar (red) are represented by dotted lines and standard deviations are observed between the full lines. Significant between-group differences are observed in the shaded region.

During CW, FOs decreased ankle dorsiflexion angle from 0 to 82% of the stance phase (%SP) (*P*<0.001) (see Fig 2A) and gastrocnemius medialis EMG amplitude at 51%SP (*P* = 0.021) (see Fig 3A, respectively. FOs also increased knee extension angle from 62 to 75%SP (*P* = 0.026) (see Fig 2A), gluteus medius EMG amplitudes from 17 to 20%SP (*P* = 0.001) (see Fig 3A) and tibialis anterior EMG amplitudes from 0 to 1%SP (*P* = 0.010) (see Fig 3A), respectively. No effect on knee frontal and transverse angles as well as vastus lateralis, gastrocnemius lateralis and peroneus longus EMG amplitudes were observed (*P*>0.05). During FW, FOs decreased ankle dorsiflexion angle from 0 to 65%SP (*P* = 0.001) (see Fig 2B). No effect on knee kinematics and EMG amplitudes were observed (*P*>0.05).

#### FOs with a lateral bar

During CW, FOs with a lateral bar decreased ankle dorsiflexion angle from 0 to 100%SP (*P* <0.001) (see Fig 2C), knee internal rotation angle from 6 to 18%SP (*P* = 0.045) (see Fig 2C) and peroneus longus EMG amplitudes from 47 to 49%SP (*P* = 0.012) (see Fig 3C), respectively. FOs with a lateral bar also increased knee extension angle from 85 to 100%SP (*P* = 0.020) (see Fig 2C), vastus lateralis EMG amplitudes from 20 to 28%SP (*P*<0.001) (see Fig 3C) and gluteus medius EMG amplitudes from 16 to 17%SP (*P* = 0.007) (see Fig 3C), respectively. No effect on knee frontal plane kinematics as well as gastrocnemius lateralis, gastrocnemius medialis and tibialis anterior EMG amplitudes (P>0.05) were observed. During FW, FOs with a lateral bar decreased ankle dorsiflexion angle from 0 to 100%SP (*P*<0.001) (see Fig 2D). No effect on knee kinematics and EMG amplitudes were observed (*P*>0.05).

## Discussion

The objectives of this study were to compare the kinematic and EMG effects of FOs with and without a lateral bar with a control condition (FOs with a lateral bar vs shoes only and FOs without a lateral bar vs shoes only) during walking in individuals with cavus feet and determine if their effects change at a fast speed. One of the main observed effect was a less dorsiflexed ankle (or more plantarflexed) when walking with FOs with and without a lateral bar compared to their respective control condition. This result is consistent with a previous study that found increased ankle plantarflexion [31] when wearing FOs. Due to the thickness of the shell, the heel was elevated of 3 mm which could explain the less dorsiflexed (or more plantarflexed) ankle angles when wearing orthoses during walking. The increased tibialis anterior EMG amplitudes at initial foot contact (FOs without a lateral bar only) could represent an attempt of the participants to increase the ankle dorsiflexion angle prior to the initial contact. Surprisingly, no changes in gastrocnemius medialis and lateralis EMG amplitudes were observed. During CW, reductions in ankle dorsiflexion when wearing both types of FOs were coupled with a greater knee extension during the second half of the stance phase and may perhaps represent a stiffer gait pattern when wearing orthoses. In general, wearing both types of FOs increased EMG amplitudes (i.e. gluteus medius, vastus lateralis and tibialis anterior) compared to their respective control condition during the beginning of the stance phase. It is unknown to what extent these EMG changes will translate into clinical effects but are likely caused by the kinematic changes aforementioned.

Concerns have been raised pertaining to the validity of using FOs designed based on Root theory [32]. The FOs with no bar used in this study were based on Root theory and are commonly prescribed by 72% of podiatrists [33]. In order to ensure the clinical applicability of the results, the effects on the biomechanics of the lower limb of the most commonly prescribed FOs in clinical practice, and to which was added a lateral bar, were compared to a control condition. According to the subtalar joint axis location and rotational equilibrium theory of foot function [34], a force acting laterally to the subtalar axis of rotation creates an external pronation moment on the ankle joint complex. Adding lateral bars to FOs should decrease the eversion moments at the ankle joint complex and thus the pronator muscles activity. FOs with a lateral bar decreased peroneus longus EMG amplitudes during the midstance phase (during CW) compared to the control condition, which was not observed for FOs. These decreased EMG amplitudes could perhaps have important clinical implications in individuals with musculoskeletal pathologies such as chronic ankle instability and peroneal tendinopathy. However, caution is suggested when interpreting this result in clinical contexts as the threshold above which the EMG changes will translate into significant improvements for the patients has yet to be determined. As the contour of the FOs in the arch area has the opposite aim of a lateral bar (i.e. increase the supinatory moments), using a thinner and more flexible material to fabricate the FOs with a lateral could perhaps enhance their effects on the biomechanics of the lower limbs. Also, FOs with a lateral rearfoot posting and a lateral heel expansion have been reported to decrease pain and plantar pressure in individuals with cavus feet [35, 36]. Further studies are needed to investigate the relationship between the decreased plantar pressure and the kinematic and EMG changes to ultimately determine the FOs that will translate into the greatest clinical benefits for individuals with painful cavus feet.

The effects of both FOs compared to their respective control condition on the biomechanics of the lower limb were different during walking, which is consistent with the results of a previous study [11]. In that study, FOs with a lateral bar decreased peroneus longus and gastrocnemius lateralis EMG amplitudes and FOs without a lateral bar decreased tibialis anterior EMG amplitudes during comfortable walking compared to a control condition for individuals with “normal” feet according to the FPI. A similar study observed increased gastrocnemius lateralis EMG amplitudes with FOs compared to a control condition, and no difference for FOs with a lateral bar during the stance phase of fast walking for individuals with cavus feet [12]. Consistent with our results, trivial or non-significant changes in EMG amplitudes for any muscle when wearing FOs with and without a lateral bar were observed during FW [12]. As the biomechanical effects of FOs with and without a lateral bar were not directly compared, it is not clear to what extent their effects are different.

Contrary to our hypothesis, the results of this study suggest that the effects of FOs with and without a lateral bar on the biomechanics of the lower limb, especially for the knee and EMG outcomes, are not more pronounced at a faster walking speed. In fact, only changes in ankle dorsiflexion angles were observed during FW. As walking at a faster speed increases the biomechanical demand to the lower limbs compared to a slower speed [37], the orthoses may need to provide more force to the feet to achieve the same level of biomechanical effects. If confirmed, the results of previous and future studies should be compared with caution when the participants walk at different speeds. Also, clinicians should consider the walking speed of their patients when extrapolating the potential biomechanical effects of the prescribed FOs. For example, older individuals walk slower than younger counterparts [38].

This study is novel as it is the first to simultaneously quantify the effects of different types of FOs on the kinematics and EMG of individuals with cavus feet during walking at CW and FW. It will help clinicians and researchers to better understand the effects of FOs on the biomechanics of the lower limbs. The main limitation of this study is the inability to directly compare the two types of FOs as the biomechanical data were collected during two experimental sessions. Reeves et al. [39] reported a good between-session reliability of the EMG data of the peroneus longus muscle when using ultrasound to guide the electrode placement. This protocol could be used in future studies to compare the effects of FOs with and without a lateral bar on peroneus longus EMG amplitudes. However, the between-session reliability of the EMG data of other lower limb’s muscles when using this protocol is still unknown. Another limitation is that frontal and transverse ankle angles were not quantified in this study. Thus, changes in these variables could be present but not observed in our study. Also, the ankle moments could not be quantified. Also, we suggest interpreting the results of this study considering the small sample size. Finally, although the participants walked slower when wearing FOs with a lateral bar compared to shoes at CW, we are confident that the observed effects are not due to walking speed as the difference was small (0.04 m/s).

## Conclusions

During walking, FOs with and without a lateral bar decreased ankle dorsiflexion angles and increased lower limb’s EMG amplitudes during early stance compared to no FOs. FOs with a lateral bar decreased peroneus longus EMG amplitudes in individuals with cavus feet which could have important clinical implications. Further studies assessing the ankle kinematics and kinetics effects of these FOs are needed to improve our understanding of their mechanism of action and inform future efficacy trials for individuals with painful cavus feet. Finally, no effect on peroneus longus EMG amplitudes were observed at a fast speed.
